# Increased TREM-2 expression on the subsets of CD11c^+^ cells in the lungs and lymph nodes during allergic airway inflammation

**DOI:** 10.1038/s41598-017-12330-6

**Published:** 2017-09-19

**Authors:** Sannette C. Hall, Devendra K. Agrawal

**Affiliations:** Department of Clinical and Translational Science, Creighton University School of Medicine, Omaha, NE USA

## Abstract

Dendritic cells (DCs) are professional APCs that traffic to the draining lymph nodes where they present processed antigens to naïve T-cells. The recently discovered triggering receptor expressed on myeloid cells (TREM)-2 has been shown to be expressed on DCs in several disease models, however, its role in asthma is yet to be elucidated. In the present study, we examined the effect of allergen exposure on TREM-2 expression in the airways and on DC subsets in the lung and lymph nodes in murine model of allergic airway inflammation. Sensitization and challenge with ovalbumin reproduced hallmark features of asthma. TREM-2 mRNA expression in the whole lung was significantly higher in the OVA-sensitized and -challenged mice which was associated with increased protein expression in the lungs. Analysis of CD11c^+^MHC-II^hi^ DCs in the lung and draining lymph nodes revealed that allergen exposure increased TREM-2 expression on all DC subsets with significantly higher expression in the lymph nodes. This was associated with increased mRNA expression of Th2 and Th17 cytokines. Further analyses showed that these TREM-2^+^ cells expressed high levels of CCR-7 and CD86 suggesting a potential role of TREM-2 in mediating maturation and migration of DC subsets in allergic airway inflammation.

## Introduction

Asthma is a chronic disorder of the conducting airways which involves the interplay of multiple genetic and environmental factors. It is characterized by reversible airway obstruction, cellular infiltration, airway inflammation and airway remodeling^1,2^. Dendritic cells (DCs) are professional antigen presenting cells (APCs) phagocytosing antigens that enter the lungs and traffic to the draining lymph nodes where they present processed antigens to T-cells driving an inflammatory Th2-response in atopic individuals. Selective expansion of Th2 cells results in the secretion of potent mediators that promote airway eosinophilia, goblet cell metaplasia and airway hyperresponsiveness (AHR)^1–4^.

Pulmonary DCs are a heterogeneous population of cells which differ phenotypically based on the expression of various cell surface markers^3^. CD11c^+^ DCs residing in the lung and lymph nodes are divided into two major subsets based on their expression of CD11b and CD103; CD11b^+/hi^CD103^−^ (referred to as CD11b^hi^) and CD11b^−/lo^CD103^+^ (referred to as CD103^+^)^5^. Lung resident CD11b^hi^ DCs reside beneath the airway epithelium, secrete high levels of chemokines and can sustain allergic inflammation during the challenge phase. CD103^+^ DCs are associated with the airway epithelium and express langerin^5,6^. There is still some debate as to which phenotype is immunogenic, promoting a Th2 response, and which is tolerogenic/regulatory, promoting a Th1/Treg response^7–11^.

Mature DCs express CC chemokine receptor 7 (CCR-7) which plays a crucial role in DC migration to secondary lymphoid organs. CCR-7 interacts with chemokines CCL19 and CCL21, which are responsible for guiding DCs from the lung to the draining lymph nodes where they can polarize naïve T-cells^12–14^. DC maturation can be induced by several signaling pathways, such as TLR, Fc receptors and DAP-12 mediated -signaling, based on the activating signal^15^.

The recently discovered triggering receptor expressed on myeloid cells (TREM)-2 belongs to a family of cell surface receptors that mediate signaling via associations with the DAP-12 adaptor protein^16^. TREM-2 is expressed on DCs, microglia and macrophages and was originally described as an anti-inflammatory mediator^17–19^. However, recent studies have shown that the TREM-2/DAP-12 pathway can provide both anti-inflammatory and pro-inflammatory signals based on the micro-environment. TREM-2 expression was found to be upregulated on bronchoalveolar lavage fluid cells of patients with pulmonary sarcoidosis^20^, and its deficiency on alveolar macrophages resulted in augmented bacterial clearance, decreased bacteremia and improved survival compared to wild type animals^21^. Viral and bacterial infections, which are known to exacerbate inflammation and impair the lung response in respiratory diseases like asthma, have been associated with increase intracellular and cell surface levels of TREM-2 on macrophages^22,23^. Studies using human DCs have shown that TREM-2 activation, via the DAP-12 pathway, promoted upregulation of CCR-7, partial DC maturation and DC survival through activation of protein tyrosine kinase (PTK) and extracellular signal-regulated kinase (ERK)-mediated signaling^24^. In the intestine, TREM-2 was shown to contribute to mucosal inflammation during colitis with TREM-2^−/−^ DCs displaying lower production of inflammatory cytokines in response to TLR ligands^25^.

Although TREM-2 provides both activating and inhibitory signals in several disease models, little is known about its expression in the airways and it is still unclear if the receptor plays a role in the pathogenesis of asthma. Given that TREM-2 has been shown to drive inflammation in other disease models, we hypothesized that TREM-2 might play a role in the onset and progression of allergic airway inflammation. Here, we examined the effect of allergen sensitization and challenge on TREM-2 expression in the airways and on DC subsets isolated from the lungs and mediastinal lymph nodes in a murine model of allergic airway inflammation.

## Results

### Sensitization and challenge with ovalbumin reproduced hallmark features of asthma

**Figure 1:**
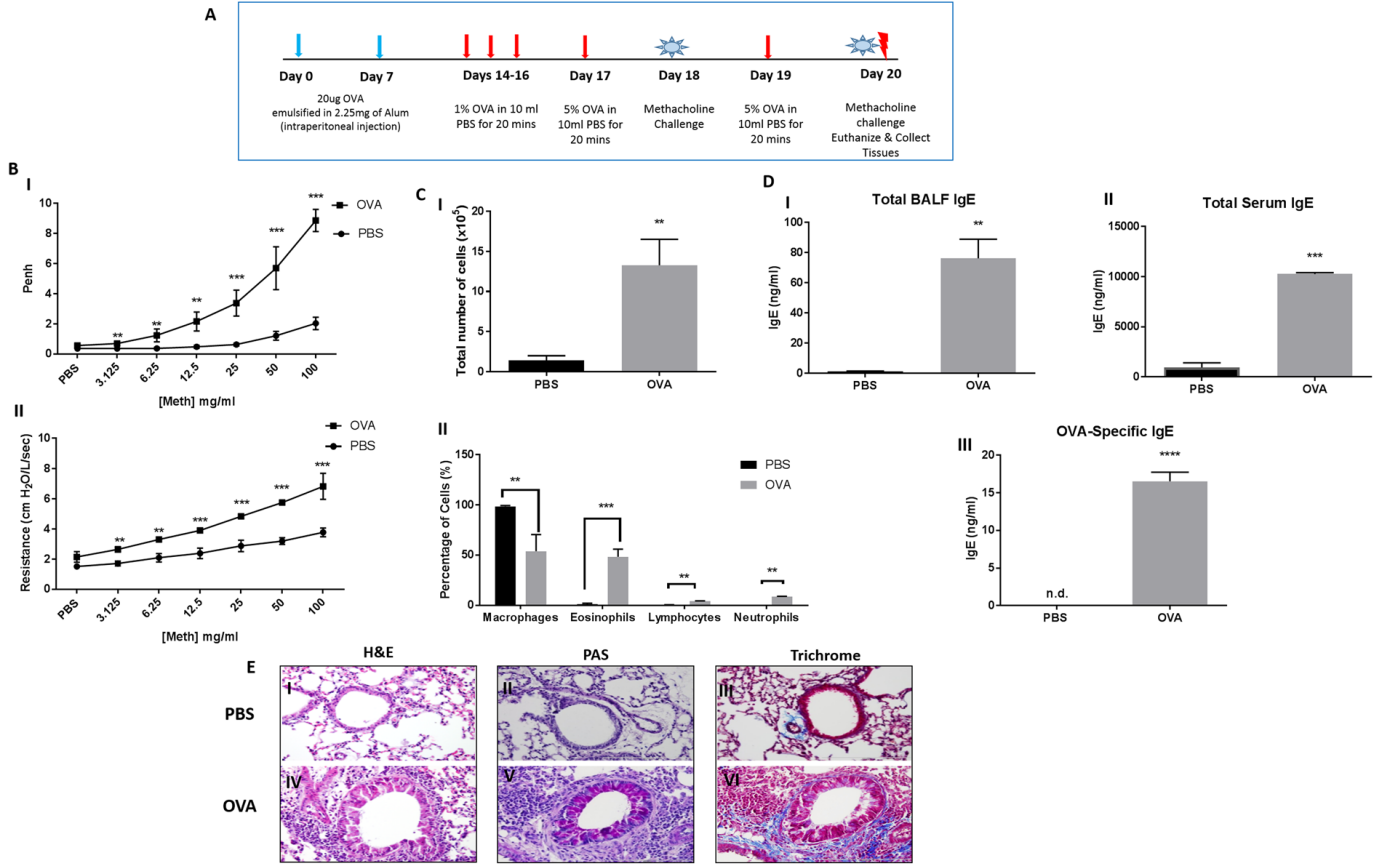
OVA-sensitization and challenge reproduced hallmark features of allergic airway inflammation. (**A**) Mice were divided into 2 groups and sensitized and challenged with either ovalbumin or sterile PBS as described in *Materials and Methods*. (**B**) Airway response to methacholine measured non-invasively (I) and invasively using anesthesia, tracheal intubation and mechanical ventilation (II) for OVA-sensitized and challenged as well as PBS control groups. (**C**)Total (I) and differential cell count (II) of inflammatory cells in bronchoalveolar lavage fluid (BALF) from PBS and OVA-sensitized and challenged mice. Lungs were lavaged with PBS, cells collected, counted, immobilized on glass slides and stained. A total of 300 cells were counted and characterized into each type of leukocyte based on morphological analyses. (**D**) Total IgE in BALF (I) and serum (II) and OVA-specific IgE in serum (III) were measured using ELISA. (**E**) Hematoxylin and Eosin (H&E) staining showing differences in lung morphology (I and IV), Periodic acid-Schiff (PAS) reaction for mucus secretion - pink coloration indicates a positive stain (II and V), Masson trichrome staining for collagen deposition - blue coloration indicates a positive stain (III-VI). Data are presented as the mean ± SEM and represent at least four independent studies. (n = 6–18 mice per group; **p < 0.01; ***p < 0.001; n.d. – not detected).

To study the effects of allergen exposure on TREM-2 expression, BALB/c mice were sensitized and challenged with ovalbumin or sterile PBS as described in Fig. 1A. OVA-sensitized and challenged mice displayed significantly higher airway response to all concentrations of methacholine (3.125 mg/ml–100 mg/ml) as determined non-invasively using whole body plethysmography and invasively using anesthesia, tracheal intubation and mechanical ventilation to determine specific airway resistance (R_L_) (Fig. 1B). Following euthanasia, blood and BALF cells were collected. Analysis of BALF revealed that OVA-sensitized and challenged mice showed greater total number of leukocytes (Fig. 1CI) as well as significantly higher percentages of eosinophils, neutrophils and lymphocytes when compared to the PBS controls (Fig. 1CII). OVA-sensitization and challenge resulted in significant increases in total IgE levels in BALF and serum when compared to the PBS-sensitized and challenged groups (p < 0.01). When we analyzed OVA-specific IgE in the serum, OVA-sensitized and challenged groups had significantly higher levels of OVA-specific IgE, which was not detected in control groups (Fig. 1DI-III). To visualize morphological changes in the airways, lung sections were stained with hematoxylin and eosin, periodic acid-Schiff (PAS) reaction and Masson’s trichrome stain (Fig. 1E). Lung sections from mice sensitized and challenged with OVA showed observable structural changes including narrowing of the lumen and increased cellular infiltration (Fig. 1EIV-VI), increased mucus secretion (Fig. 1EV) and collagen deposition (Fig. 1EVI) which were absent in PBS-sensitized and challenged groups (Fig. 1I-III). These results show that our mouse model can generate hallmark features of allergic asthma.

### Sensitization and challenge with OVA increases TREM-2 expression in the airways

**Figure 2:**
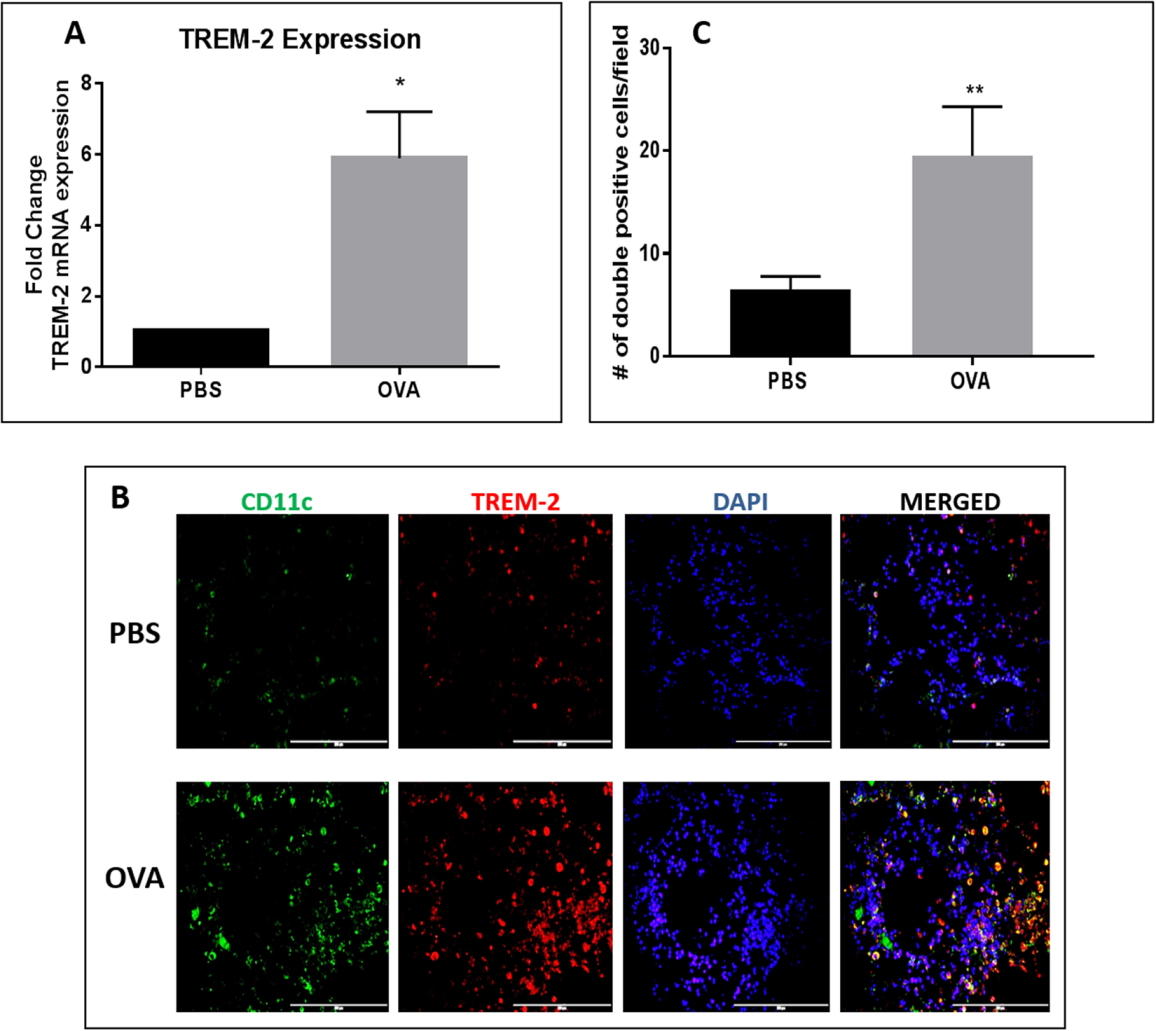
TREM-2 expression in whole lung tissue from OVA-sensitized and challenged mice as well as PBS controls. (**A**) Total RNA was isolated from the lungs, converted to cDNA and subjected to qRT PCR for TREM-2 mRNA expression. (**B**) TREM-2 protein expression in whole lung sections from PBS and OVA-sensitized and challenged mice. Sections were stained using hamster anti-CD11c and goat anti-TREM-2 primary antibodies and rabbit anti-goat Alexa Fluor (red) and rabbit anti-hamster FITC (green) secondary antibodies (Mag ×40). (**C**) Quantification of the number of CD11c^+^ and TREM-2^+^ cells. Data are presented as the mean ± SEM and represent at least three independent studies. (n = 3–6 mice per group; *p < 0.05; ***p < 0.001).

To ascertain whether TREM-2 was expressed in the airways, we first examined mRNA expression of TREM-2 in the lungs isolated from OVA-sensitized and challenged mice as well as PBS control groups. Using RT-PCR, it was found that TREM-2 mRNA transcripts were significantly upregulated in OVA-sensitized and challenged groups when compared to control mice (Fig. 2A) (p < 0.05). To determine if TREM-2 was expressed on CD11c^+^ cells in the airways, fixed sections were stained for dual expression of CD11c (pan DC marker) and TREM-2 and co-localization determined by fluorescent microscopy. Lung sections from OVA-sensitized and challenged groups had more than twice the number of cells expressing both CD11c and TREM-2, as indicated by yellow-orange staining, when compared to PBS controls (Fig. 2B,C), suggesting that TREM-2 might contribute to the inflammatory response associated with allergic asthma.

### OVA-sensitization and challenge generates distinct subsets of CD11c^+^ cells in the airways and lymph nodes

**Figure 3:**
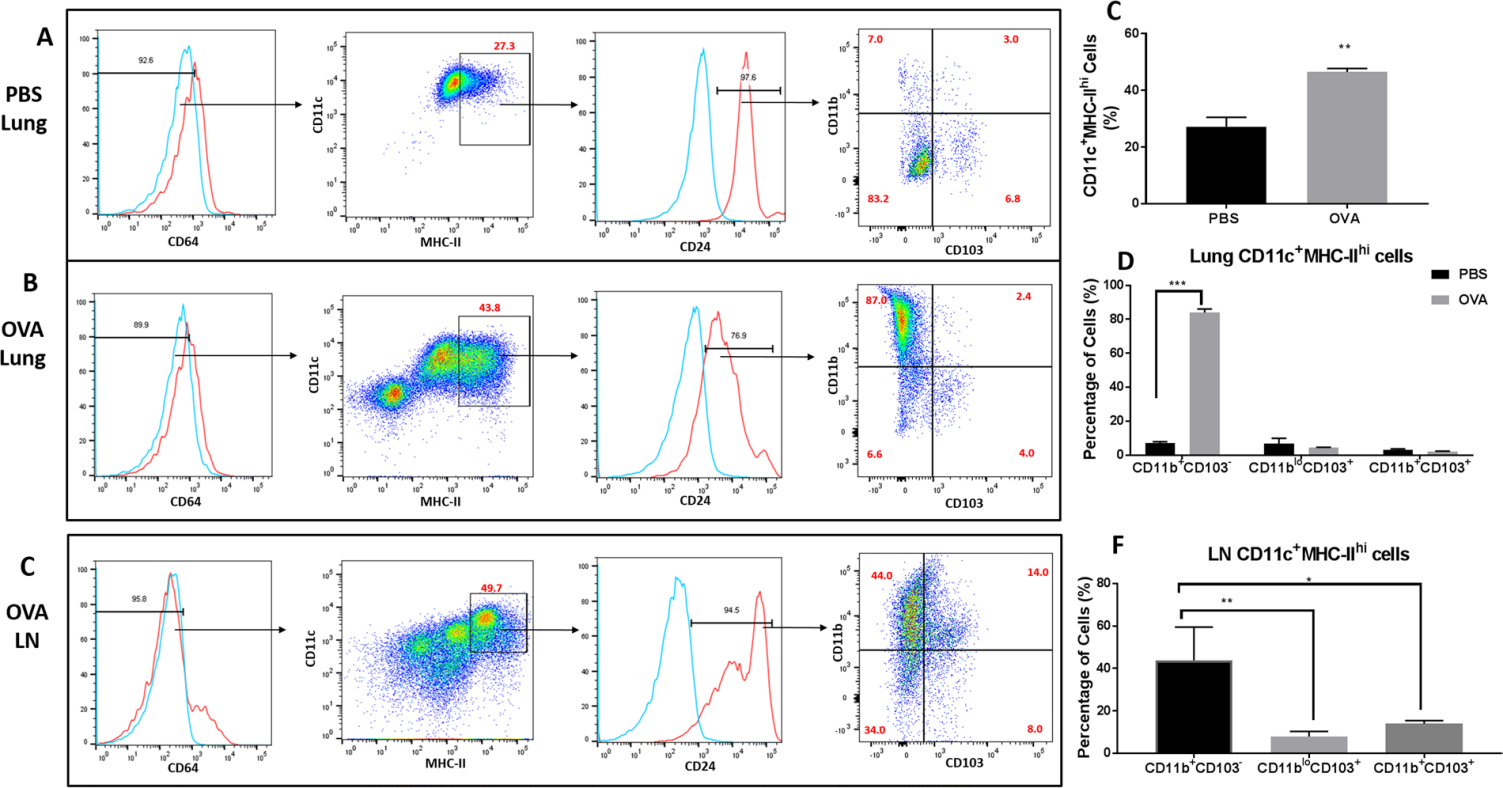
Sensitization and challenge with ovalbumin generates distinct subsets of CD11c^+^ cells in the lungs and lymph nodes. (**A**,**B)** Lungs and lymph nodes (**E**) from OVA-sensitized and challenged and PBS mice were isolated and analyzed using FACS to determine the phenotype of CD11c^+^ cells. Cells were gated based on expression of CD64, MHC-II, CD24, CD11b and CD103. (**C**,**D** and **F**) Quantitative analyses of percentage expression of CD11c ^+ ^MHC-II subsets in the lungs (**C**) and percentage expression of each subset in the lungs (**D**) and lymph nodes of OVA-sensitized and challenged mice (**F**). Data are presented as the mean ± SEM and represent at least four independent studies. (n = 5–10 mice per group; * p < 0.05; **p < 0.01; ***p < 0.001).

Given that there was colocalization of TREM-2 with CD11c in the airways, our next step was to determine the phenotype of CD11c^+^ cells generated after exposure to ovalbumin. Lung lobes from PBS and OVA-sensitized and -challenged mice were isolated, processed and analyzed by flow cytometry to determine the phenotype of DCs in the airways and mediastinal lymph nodes. After gating out debris and doublets, live cells were selected and gated based on expression of CD64, CD24 and high levels of MHC-II to determine mature DCs and eliminate alveolar macrophages (macrophages are CD64^+^ and CD24^−^). For our analyses, we selected the subsets of cells that were CD64^−^ (blue histogram), CD11c^+^MHC-II^hi^ and CD24^+^ (red histogram). These cells were further analyzed based on the expression of CD11b and CD103 (Fig. 3A,B). In the airways, it was found that the OVA-sensitized and -challenged groups had greater percentage of mature CD11c^+^MHC-II^hi^ cells when compared to the PBS group (Fig. 3C). Further analyses of this subset of cells revealed that there were four populations of CD11c^+^ cells in the airways: CD11b^+^CD103^−^ (CD11b^hi^), CD11b^lo/−^CD103^+^ (CD103^+^), CD11b^+^CD103^+^ and a subset of cells that did not express either markers (CD11b^−^CD103^−^) (Fig. 3A,B). Most of the cells isolated from the PBS-sensitized and challenged groups were negative for both CD11b and CD103. In the OVA-sensitized and -challenged groups however there were significant increases in the percentages and total number of cells which were CD11b^+^CD103^−^ (CD11b^hi^) (Fig. 3B and D). As seen in the airways, four populations of CD11c^+^ cells were identified in the mediastinal lymph nodes: CD11b^+^CD103^−^ (CD11b^hi^), CD11b^−^CD103^+^ (CD103^+^) CD11b^+^CD103^+^ and CD11b^−^CD103^−^ (Fig. 3E). Interestingly, there was an increase in the percentage of CD11b^+^CD103^+^ when compared to the lungs and a decrease in the CD11b^hi^ subset. Overall, the CD11b^+^CD103^−^ (CD11b^hi^) subsets had significantly higher percentage of cells when compared to the CD11b^−^CD103^+^ (CD103^+^) (p < 0.01) and CD11b^+^CD103^+^ subset (p < 0.05) (Fig. 3F). When we examined DC populations in the lymph nodes of PBS-sensitized and challenged mice, very few cells were found (data not shown). These data show that CD11c^+^ cells in the lungs and lymph nodes consists of a heterogeneous population of cells with increased CD11b subsets in the airways and increased double positive subsets in the lymph nodes.

### CD11c^+^ cells in the lung and lymph nodes express TREM-2

**Figure 4:**
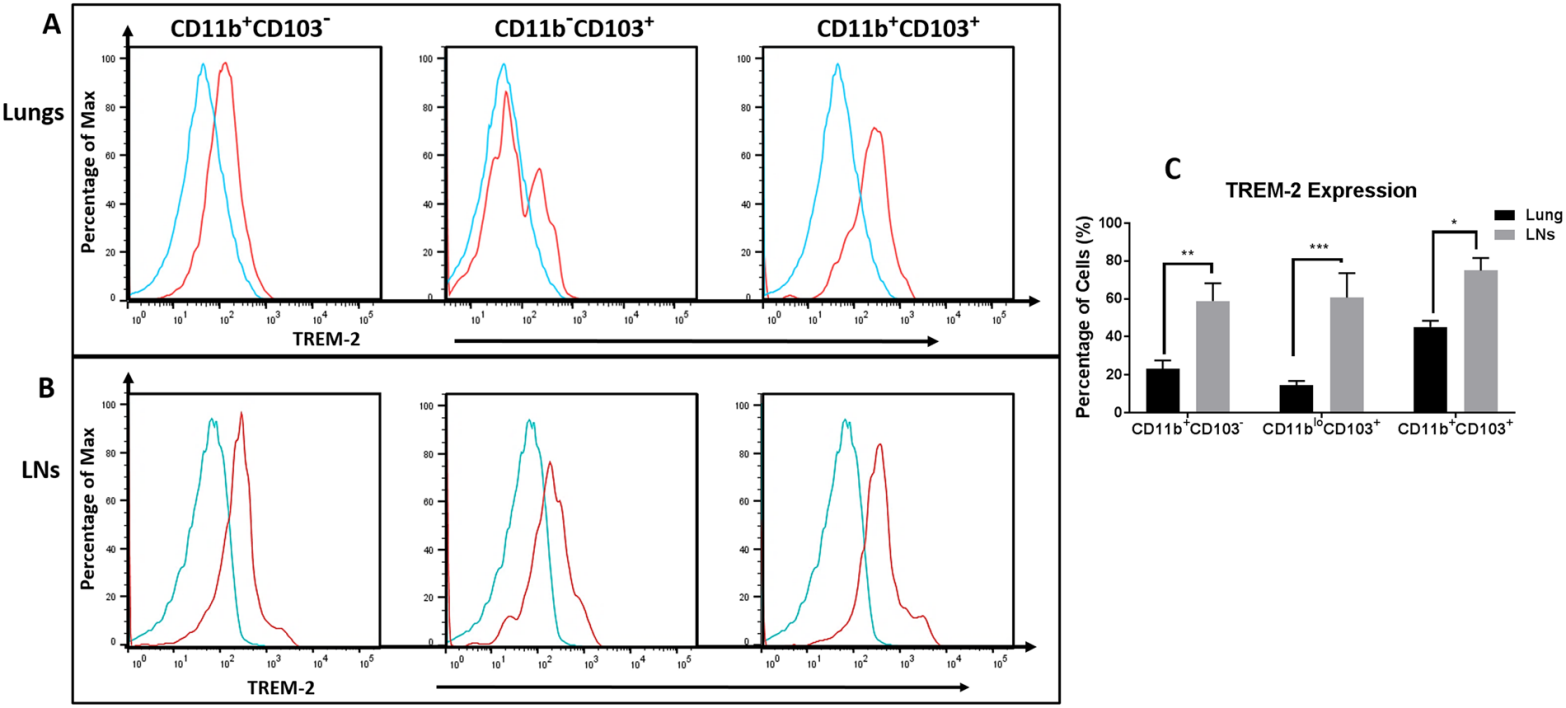
Increased TREM-2 expression on subsets of cells isolated from the lungs and lymph of OVA-sensitized and challenged mice. (**A**,**B**) The three subsets of cells isolated from the lungs and lymph nodes were further analyzed for expression of TREM-2 by FACS. Gating was done based on expression of CD64, MHC-II, CD24, CD11b, CD103 and TREM-2 expression analyzed on these cells. Blue histograms - Isotype controls, red histograms - stained samples. (**C**) Quantitative analysis of percentage TREM-2 expression in the lungs and lymph nodes. Data are presented as the mean ± SEM and represent at least four independent studies. (n = 5–10 mice per group; *p < 0.05; **p < 0.01; ***p < 0.001).

The immunofluorescence data showed that TREM-2 was found to be expressed on CD11c^+^ cells in the airways and our previous results showed that this is a heterogeneous population. To confirm that TREM-2 was expressed on the subsets identified, each population was further analyzed for TREM-2 expression by FACS. All subsets of cells isolated from the lungs and lymph nodes express some amount of TREM-2. In the airways, TREM-2 expression was found to be higher on the subsets of cells isolated from OVA-sensitized and challenged mice (Fig. 4A and C) when compared to the PBS controls (data not shown). Of the three DC subsets identified, TREM-2 expression was highest on the double positive (CD11b^+^CD103^+^) and CD11b^hi^ subsets (Fig. 4C). Similar results were found on the subsets of cells isolated from the mediastinal lymph nodes (Fig. 4B,C), confirming that CD11c^+^ cells in the lungs and lymph nodes express TREM-2. When we compared TREM-2 expression among the subsets in the lungs and lymph nodes, it was found that there was significantly increased TREM-2 expression on all subsets of cells in the lymph nodes when compared to those in the airways (p < 0.05). The CD11b^hi^ and CD103^+^ subsets had the greatest overall increase in TREM-2 expression in the lymph nodes, with approximately twice the percentage of TREM-2^+^ cells (p < 0.01 and p < 0.001 respectively) when compared to the lungs (Fig. 4C). Results suggest that there is increased TREM-2 expression on CD11c^+^ cells migrating from the lungs to the lymph nodes after exposure to ovalbumin.

### TREM-2^+^cells express higher CCR-7 and CD86 when compared to their TREM-2^−^ counterparts

**Figure 5:**
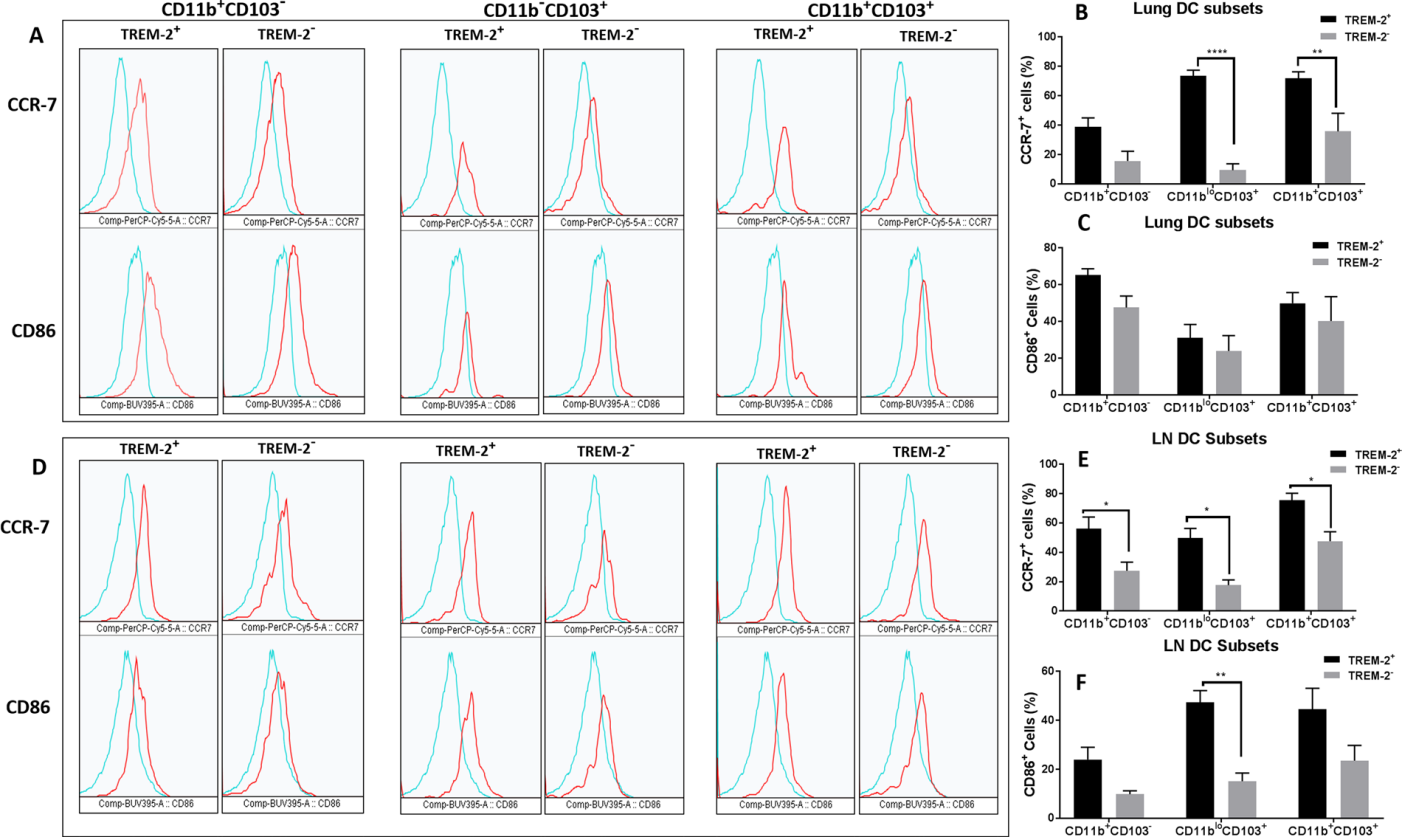
Increased CCR-7 and CD86 expression on TREM-2^+^ subset of cells isolated from the lung and lymph nodes of OVA-sensitized and challenged mice. (**A**–**F**) Lung (**A**) and lymph node (**D**) TREM-2^+^ and TREM-2^−^ subsets were analyzed for expression of CCR-7 and CD86 using FACS. Blue histograms - Isotype controls, red histograms - stained samples. (**B**,**C** and **E**,**F**) Quantitative analyses of CCR-7 and CD86 expression on TREM-2 subsets isolated from the lung and lymph nodes. Data are presented as the mean ± SEM and represent at least four independent studies. (n = 5–10 mice per group; *p < 0.05; **p < 0.01; ***p < 0.001).

Given that we saw increased TREM-2 expression on subsets of cells in the lymph nodes and CCR-7 is crucial for migration of dendritic cells, we next determined expression of CCR-7 and CD86 on TREM-2 positive and negative subsets isolated from the lungs and lymph nodes of OVA-sensitized and challenged mice. In the lungs, TREM-2 positive cells had greater CCR-7 and CD86 expression when compared to their TREM-2^−^ counterparts (Fig. 5A–C). CCR-7 expression was highest on the CD103^+^ and CD11b^+^CD103^+^ TREM-2^+^ subsets (p < 0.001 and p < 0.01 respectively) with no significant differences seen in the CD11b^hi^ TREM-2^+^ subsets (Fig. 5B). Though not significant, CD86 expression was higher on TREM-2^+^ subsets with the CD11b^hi^ subset of cells having the greatest CD86 expression (Fig. 5C). In the lymph nodes, both CCR-7 and CD86 expression was increased on all TREM-2^+^ subset of cells (Fig. 5D–F). CCR-7 expression was significantly higher on all three TREM-2^+^ subsets when compared to the TREM-2^−^ cells (p < 0.05), with the double positive subset having the greatest CCR-7 expression (Fig. 5E). These data show that there is increased CCR-7 and CD86 expression on TREM-2^+^ cells in the lungs and lymph nodes highlighting a potential role of TREM-2 in the migration and maturation of CD11c^+^ cells.

### Increased TREM-2 expression in the lymph nodes is associated with increased Th2 and Th17 responses

**Figure 6:**
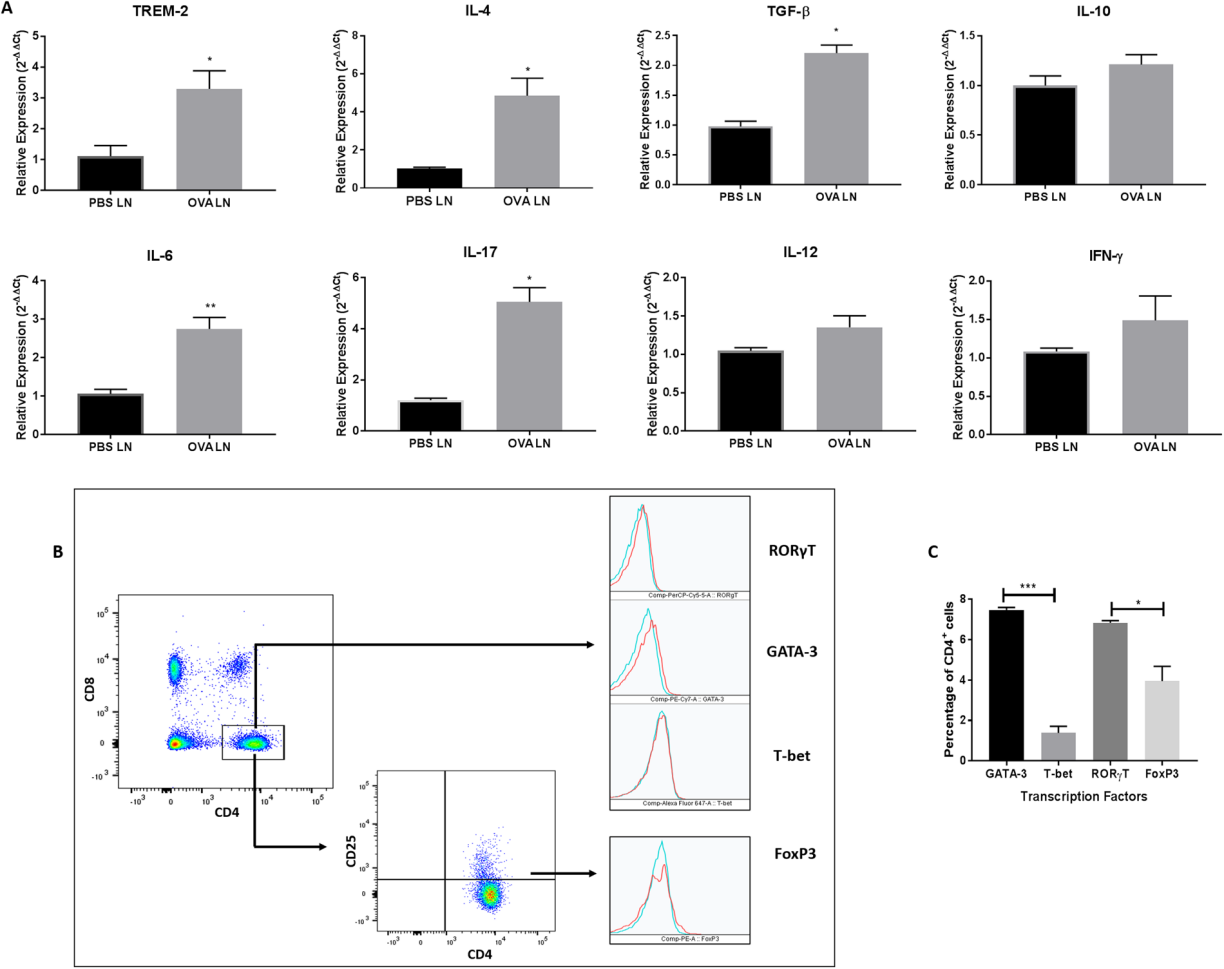
Increased TREM-2 expression is associated with increased Th2 and Th17 responses in the lymph nodes of OVA-sensitized and challenged mice. (**A**) Total RNA was isolated from the lymph nodes of OVA-sensitized and challenged and PBS groups, converted to cDNA and subjected to qRT PCR for mRNA expression of TREM-2, IL-4, IL-6, IL-17, IL-10, IL-12, TGF-β and IFN-γ. (**B**) Lymph nodes from OVA-sensitized and challenged mice were isolated and analyzed using FACS to determine the phenotype of CD4^+^ cells. Cells were gated based on expression of CD4 and CD8. The CD4^+^ subset was further analyzed for the expression of RORγt, GATA-3, T-bet and FoxP3. (**C**) Quantitative analyses of percentage expression of each transcription factor in the lymph nodes of OVA-sensitized and challenged mice. Data are presented as the mean ± SEM and represent at least three independent studies. (n = 4–6 mice per group; *p < 0.05; **p < 0.01; ***p < 0.001).

To determine the T helper cell subsets that were being generated by increased TREM-2 expression on DCs migrating to the lymph nodes, we examined mRNA expression of Th1, Th2, Th17 and Treg cell cytokine secretion using RT PCR as well as expression of transcription factors using FACS in the mediastinal lymph nodes. Our results showed significant increases in mRNA expression of IL-4, IL-6, IL-17 and TGF-β in the lymph nodes isolated from OVA-sensitized and challenged mice compared to control groups (p < 0.05; Fig. 6A). Of the cytokines examined, IL-4 and IL-17 had overall greatest increase Relative expression of IL-10, IL-12 and IFN-γ were only marginally higher in the OVA-sensitized and challenged groups and showed no significant differences when compared to the controls (Fig. 6A). Analysis of FACS data revealed that approximately 30% of cells isolated were CD4^+^ T-cells. Further analyses of these cells showed increased expression of GATA-3 when compared to T-bet (p < 0.001) and increased RORγt expression when compared to FoxP3 (p < 0.05; Fig. 6B-C). These results suggest that increased TREM-2 expression on DCs in the lymph nodes play a role in driving Th2 and Th17 responses.

## Discussion

Dendritic cells are the primary antigen presenting cells that control the initiation of antigen-induced immune response in the airways. Lung DCs are a heterogeneous population of cells which express CD11c and are further divided into two major subsets based on the expression of CD11b and CD103^5,6^. Several studies have shown that the phenotype of lung DC subsets plays a role in determining the nature of the immune response to inhaled antigens. Results from these studies suggest that both CD11b^+^ and CD103^+^ DCs can drive inflammation based on the dose and route of administration of the allergen as well as the duration of the antigen-sensitization and challenge protocol^7–11^.

In the steady state, we identified three populations of cells in the airways: CD11b^+^CD103^−^ (CD11b^hi^) and CD11b^−^CD103^+^ (CD103^+^), as reported in the literature, as well as another subset of cells that expressed both CD11b and CD103 (CD11b^+^CD103^+^). Exposure to ovalbumin resulted in an increase in the percentage of cells that were CD11b^hi^ and slight decreases in the percentage of cells that were CD11b^+^CD103^+^ and CD103^+^, though total cell numbers of each subset was higher in the OVA-sensitized and challenged groups. It has been shown that exposure to allergen leads to rapid recruitment of monocyte-derived CD11b^hi^ DCs to the airways^26,27^. CD11b^hi^ DCs also secrete a host of pro-inflammatory chemokines which mediate chemotaxis of immune cells driving allergic airway inflammation^5,6^. Additionally, CD11b^hi^ DCs have been shown to be more efficient in OVA uptake than CD103^+^ subset of cells^7^ which could account for the vast increase in this population observed in the airways. Our results, as well as the finding from other groups, suggest that exposure to ovalbumin increases CD11b^hi^ DCs in the airways and this subset of cells is possibly the one driving localized inflammation observed in the airways of these animals. As seen in the airways, three populations of DC subsets were also identified in the draining lymph nodes. When compared to the lungs, there was an increase in percentage of CD103^+^ and CD11b^+^CD103^+^ cells and a decrease in the percentage of CD11b^hi^ cells. It should be noted that total numbers of cells in this population was still the highest, further confirming that the CD11b^hi^ subset is the major contributor in the generation and progression of the inflammatory response seen in the OVA-sensitized and challenges group.

TREM-2 belongs to a family of recently discovered cell surface receptors which have been shown to play a role in fine tuning innate responses induced by TLRs^17,28^. Although its ligand is yet to be identified, TREM-2 mediates signaling via association with the DAP-12 adaptor protein^16^. It is generally regarded as an anti-inflammatory mediator but has been shown to amplify the production of pro-inflammatory cytokines and upregulate CCR7 expression, which is crucial for migration of dendritic cells to the draining lymph nodes^18,24,25,29^. Recently, TREM-2 has also been shown to be upregulated on DCs that contribute to bone destruction in a murine model of acquired cholesteatoma^30^.

Although the receptor has been implicated in a number of disease models^17,21,31,32^, its expression and function has not been clearly elucidated in allergic airway inflammation. As seen with other inflammatory conditions^20,22,25,30,31^, TREM-2 was found to be upregulated in the airways of mice exposed to OVA when compared to the control groups. In steady state, TREM-2 was expressed on all subsets of DCs identified in the lungs confirming that DCs do express TREM-2. OVA-sensitization and challenge led to upregulation of TREM-2 expression on the three subsets of CD11c^+^ cells in both the lungs and mediastinal lymph nodes. Increased TREM-2 expression in the lymph nodes suggest that TREM-2 might be involved in the mechanism driving migration of DC subsets.

Further examination of TREM-2 positive subsets in the lungs and lymph nodes revealed that these cells had greater CCR-7 expression compared to their TREM-2 negative counterparts. It is well established that upregulation of CCR-7 is critical for migration of DCs to the draining lymph nodes and DCs migrate under the influence of lymphatic chemokines CCL-19 and CCL-21. We have previously shown that OVA-sensitization and challenge resulted in increased expression of CCL-19 and CCL-21 in the T-cell zone of the cortex and upregulated CCL-21 in the afferent lymphatic vessels and high endothelial vesicles in the mediastinal lymph nodes^12^. Subsets of cells that expressed higher levels of CCR-7 were also shown to be better at migrating towards CCL-19 and CCL-21 *in vitro* ^33^. Of the three subsets of cells identified in the airways, the CD11b^hi^ subset, though the predominant phenotype, was shown to have the lowest overall CCR-7 expression. Studies have shown that while lung resident CD11b^hi^ DCs are more efficient at antigen uptake, they migrate poorly to the lymph nodes and tend to remain in the airways where they mediate the production of pro-inflammatory cytokines^7,34^. If the CD11b^hi^ lung DC population was made up of some infiltration monocyte-derived CD11b^hi^ DCs, these cells have been shown to express little-to-no CCR-7 even after stimulation^35^. Since CCR-7 is crucial for dendritic cell migration, lack of expression on a population of these cells would prevent them from migrating, thus decreasing the percentage of this phenotype observed in the lymph nodes.

Our findings suggest that increased TREM-2 expression on subsets of cells in the lymph nodes may be driving Th2 and Th17 responses given the marked increase in mRNA expression of IL-4, IL-6 and IL-17 in OVA-sensitized and challenged mice. This suggests some division of labor among the subsets of cells identified in our studies. Several studies have shown that CD11b^+^ DCs secrete a host of pro-inflammatory mediators and play a role in priming and re-stimulating effector CD4^+^ T-cells driving Th2 responses^5,6,9,11,27^. The CD103^+^ DCs have also been shown to prime Th1 as well as Th17 responses^11^. It is now well known that Th17 cytokines induce mucus cell metaplasia, neutrophil recruitment, AHR and airway remodeling^36^. The increased numbers of CD103^+^ DCs in the lymph nodes, increased TREM-2 expression on these cells as well as increased expression of IL-6, TGF-β and IL-17 highlight a potential role of these cells in driving Th17 responses in OVA-sensitized and challenged mice.

Overall, TREM-2 expression was found to be highest on the double positive subset of cells in both the lungs and lymph nodes with increased total number of these cells in the lymph nodes. This subset of cells has yet to be characterized in the airways, however, studies have shown that during inflammatory conditions, CD11b^+^ DCs can express CD103^5,37^ leading to the observed phenotype seen in our results. CD11b^+^CD103^+^ dendritic cells have been recently characterized in the intestine as *bona fide* dendritic cells that are capable of priming Th17 cells^38,39^. Given that our results have also shown that there was increased mRNA expression of Th17 associated cytokines, which are known to play a critical role in airway neutrophilia and AHR, further studies are required to determine if a similar response occurs in the airways.

The current studies were focused on TREM-2 expression on DC subsets in the lungs and lymph nodes. It is important to note that TREM-2 has been found to be expressed on other cell types in the airways, including macrophages, which may also contribute to allergic airway inflammation^22,40^. When we examined TREM-2 expression on the CD64^+^CD11c^lo^CD11b^+^ subset of cells in the airways, there was very mild expression found on these cells. More in-depth studies using macrophage-specific markers to accurately gate this population of cells with high purity would be needed to determine the exact contribution of TREM-2 expressing macrophages to the response seen in OVA-induced lung inflammation.

TREM-2 upregulation in the lungs and lymph nodes along with increased expression of transcripts for Th2 and Th17 cytokines in the lymph nodes of mice exposed to ovalbumin could indicate that the receptor is participating in the onset or progression of allergic airway inflammation. Two recent studies have implicated the receptor as a pro-inflammatory mediator in airway inflammation. Analysis of BALF from patients with asthma showed increased expression of TREM-2 which was associated with increased eosinophil and eosinophilic inflammation^41^. Another study has shown that TREM-2 was upregulated in the lungs of an experimental model of melioidosis and TREM-2 deficient mice had markedly reduced inflammation^40^. Other studies have shown that the receptor plays a role in dampening the immune response. It is, therefore, reasonable to assume that the induction of TREM-2 on DC subsets in the airways and lymph nodes could indicate an impaired defense mechanism originally aimed at decreasing rather than enhancing inflammation.

**Figure 7:**
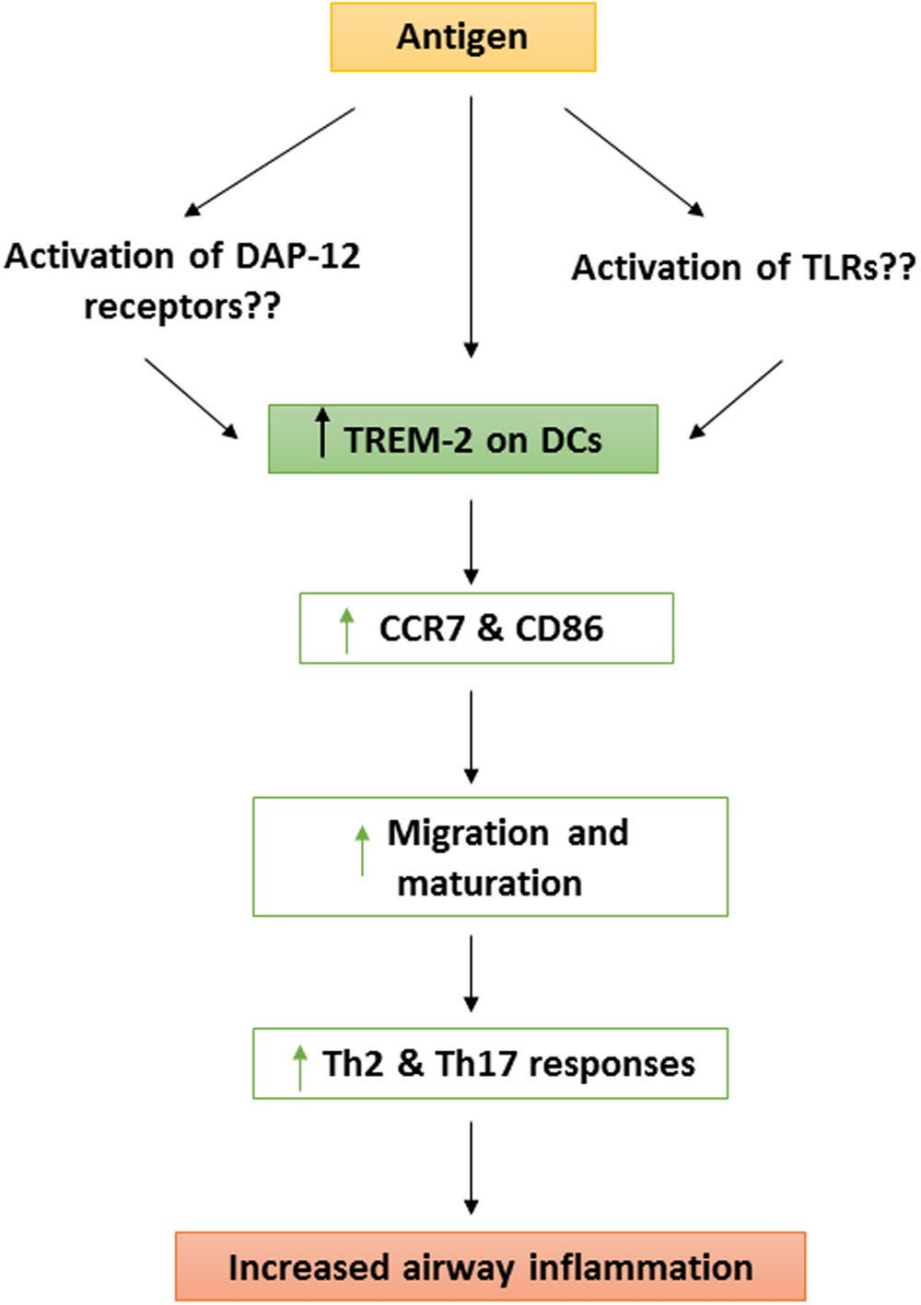
Schematic diagram of proposed role of TREM-2 in allergic airway inflammation. Exposure to antigen results in either activation of TLRs and/or DAP-12 DC receptors or directly leads to upregulation of TREM-2 on DCs. TREM-2 then amplifies these pro-inflammatory signals leading to upregulation of CCR-7 and CD86 on DC subsets. This promotes migration and maturation of these cells leading to increased antigen presentation and priming of Th2 and Th17 response, thus promoting airway inflammation.

The mechanism by which TREM-2 might be driving inflammation remains unclear. Results from existing studies seem to suggest that the cell type that expresses the receptor as well as the cytokine milieu might influence its role in inflammation. When expressed on macrophages and microglia, TREM-2 seems to play more of an anti-inflammatory role^29,42,43^. However, based on the results from our studies as well as other groups, when expressed on dendritic cells, the role seems to be reversed^25,30^. It is our speculation that the receptor could be contributing to allergic airway inflammation by amplifying pro-inflammatory signals generated by TLRs or other DAP-12 DC associated receptors ultimately resulting in increased survival, maturation and migration of DC subsets. This has been summarized in schematic diagram shown in Fig. 7.

## Materials and Methods

### Animals and Care

Five-to-six-week-old female Balb/c mice were purchased from Envigo/Harlan Laboratories (Indianapolis, IN) and maintained under specific pathogen-free conditions in the Animal Resource Facility at Creighton University. Food and water were provided *ad libitum*. The research protocol of these studies was approved by the Institutional Animal Care and Use Committee of Creighton University. All methods in the animal care and procedures in this research protocol were performed in accordance with the NIH and OLAW guidelines.

### Sensitization and Challenge of Experimental Animals

Mice were divided into sensitized and non-sensitized groups. The sensitized groups received an intraperitoneal injection of 20 μg of OVA (Sigma-Aldrich, St. Louis, MO) emulsified in 2.25 mg of Imject® alum (Thermo Fischer Scientific, Waltham, MA) on days 0 and 7. Animals were challenged with 1% OVA for 3 consecutive days (day 14–16) and 5% OVA on day 17. The non-sensitized groups received sterile PBS. Airway response to methacholine was determined non-invasively on day 18 using whole body plethysmography single chamber (Buxco® FinePoint System, Data Science International, St. Paul, MN). Animals with established AHR were challenged with 5% OVA or sterile PBS on day 19. On day 20, specific airway resistance was measured invasively under anesthesia in tracheal intubated and mechanically ventilated mice using Buxco® FinePointe RC System (Data Science International, St. Paul, MN).

### Bronchoalveolar Lavage Fluid Analysis and IgE Measurements

Immediately after euthanasia, blood was collected from the left ventricle of the heart and the separated serum was stored at −80 °C for further analysis. Bronchoalveolar lavage fluid (BALF) was collected by lavaging the lungs with 1 ml of warm PBS. The samples were then centrifuged at 400 × *g* for 10 minutes and the supernatant collected and stored at −80 °C for further analysis. The cell pellet was resuspended in PBS and total cell counts were performed using Countess Automated Cell Counter (Invitrogen, Grand Island, NY). Cells were immobilized on glass slides by cytospin centrifugation (Shandon Cytospin 4, Thermo Fisher Scientific, Waltham, MA), fixed in acetone and stained using Diff-Quik staining reagent (StatLab Medical Products, Lewisville, TX) according to manufacturer’s instructions. Differential cell counts were carried out by randomly counting 300 cells as visualized under light microscopy.

Total and OVA-specific IgE in BALF and serum were measured using commercially available ELISA (eBioscience, San Diego, CA and BioLegend, San Diego, CA) according to manufacturers’ instructions.

### Lung Histology

Lung lobes were removed, fixed in 4% formalin and embedded in paraffin. The 5 μm thin sections of the fixed tissues were rehydrated and stained using hematoxylin and eosin (Newcomer Supply, Middleton, WI), periodic acid-Schiff (PAS) reaction (Sigma-Aldrich, St. Louis, MO) and Masson’s trichrome stain (Sigma-Aldrich, St. Louis, MO) according to manufacturers’ instructions.

### Immunofluorescence

Paraffin embedded sections were stained for co-expression of TREM-2 and CD11c. Briefly, non-specific binding was blocked by incubating sections with 5% rabbit serum in PBS for 1 hour. Sections were washed twice with PBS and incubated with primary mouse antibodies to TREM-2 (1:200; abcam, Cambridge, MA) and CD11c (1:200; abcam, Cambridge, MA) overnight at 4 °C. The sections were then washed and incubated with rabbit anti-goat Alexa Fluor (1:500; abcam, Cambridge, MA) and rabbit anti-hamster FITC (1:500; ImmunoReagents, Raleigh, NC) secondary antibodies for 2 hours at room temperature. Following the final PBS washes, sections were fixed using Vectashield mounting media with DAPI (Vector Laboratories, Burlingame, CA). Cells were counted using an Olympus DP71 camera at ×40 magnification. TREM-2 was labelled in red, CD11c labelled in green, and the nuclei stained blue.

### Quantitative real time PCR

Total RNA was isolated from the lung and lymph nodes using Trizol reagent according to the manufacturer’s protocol (Sigma, St. Louis, MO, USA) and total RNA yield quantified using Nanodrop (Thermo Fisher Scientific, Waltham, MA). First-strand cDNA synthesis was performed using 1 µg total RNA with oligo dT (1 µg), 5 X reaction buffer, MgCl2, dNTP mix, RNAse inhibitor and Improm II reverse transcriptase as per Improm II reverse transcription kit (Promega, Madison, WI). Following the first strand synthesis, real time PCR was carried out using cDNA, SYBR green PCR master mix (Bio-Rad Laboratories, Hercules, CA) and forward and reverse primers for TREM-2, IL-4, IL-6, IL-10, IL-17, IL-12, IFN-γ and TGF-β (Integrated DNA Technologies, San Diego, CA) using a real-time PCR system (CFX96, BioRad Laboratories, Hercules, CA). Relative gene expression was normalized against the housekeeping gene, glyceraldehyde-3-phosphate dehydrogenase (GAPDH) and fold-change in mRNA expression determined using ^ΔΔ^Ct method. The primers used in these experiments include the following: TREM-2 Forward primer 5′-TGGTGTGGTACATCTGTGTTGAG-3′, Reverse primer 5′-GGTTGGTGTGTGGAGAATGT-3′; IL-4 Forward primer 5′-CAGAGACTCTTTCGGGCTTT-3′, Reverse primer 5′-GCATGATGCTCTTTAGGCTTTC-3′; IL-6 Forward primer 5′-CCAGAGTCCTTCAGAGAGATACA-3′, Reverse primer 5′-CCTTCTGTGACTCCAGCTTATC-3′; IL-10 Forward primer 5′-CCAAGACCAAGGTGTCTACAA-3′, Reverse primer 5′-GGAGTCCAGCAGACTCAATAC-3′; IL-12 Forward primer 5′-GGACCAAAGGGACTATGAGAAG-3′, Reverse primer 5′-CTTCCAACGCCAGTTCAATG-3′; IL-17 Forward primer 5′-TCCAGAAGGCCCTCAGACTA-3′, Reverse primer 5′-ACACCCACCAGCATCTTCTC-3′; TGF-β Forward primer 5′-GGTGGTATACTGAGACACCTTG-3′, Reverse primer 5′-CCCAAGGAAAGGTAGGTGATAG-3′; IFN-γ Forward primer 5′-ATCGGCTGACCTAGAGAAGA-3′, Reverse primer 5′-AGCCAAGATGCAGTGTGTAG-3′; GAPDH Forward primer 5′-AACAGCAACTCCCACTCTTC-3′, Reverse primer 5′-CCTGTTGCTGTAGCCGTATT-3′.

### Lung and Lymph NNode Dendritic Cell Isolation

Lung lobes and mediastinal lymph nodes were collected and perfused with dissociation enzyme mix (Miltenyi Biotech, Auburn, CA), finely chopped and incubated for 20 minutes at 37 °C. After enzymatic digestion and red blood cell lysis, samples were resuspended in MACS buffer and total number of cells counted using Countess Automated Cell Counter. Cells were incubated at 4 °C with FcR blocking reagent for 10 minutes followed by another 10-minute incubation with CD11c ultrapure microbeads (Miltenyi Biotech, Auburn, CA). The cells then underwent a positive selection using the Posseld2 program on the AutoMACS Pro Separator (Miltenyi Biotech, Auburn, CA). The CD11c^+^ fraction was collected for flow cytometry analysis.

### Flow Cytometry for Dendritic Cells

CD11c^+^ fraction was incubated with LIVE/DEAD fixable aqua dead cell stain kit (Thermo Fisher Scientific) for 30 minutes at 4 °C. After centrifugation cells were incubated with an antibody cocktail consisting of: CD11c PE/CF594, CD86 BUV395 and CD24 BUV 737 (BD Biosciences, San Jose, CA); CD11b PE/Cy7, CD103 PE and CCR-7 PerCP/Cy5.5 (eBioscience, San Diego, CA); MHCII FITC (Biolegend, San Diego, CA); CD64 Alexa Fluor 700 and TREM-2 APC (R&D Systems, Minneapolis, MN). Cells were analyzed by fluorescence activated-cell sorting (BD FACSAria™, BD Biosciences, San Jose, CA) to determine DC subsets in the lungs and mediastinal lymph nodes.

### Flow Cytometry for CD4 + T Cells

Mediastinal lymph nodes were collected and perfused with dissociation enzyme mix (Miltenyi Biotech, Auburn, CA), finely chopped and incubated for 20 minutes at 37 °C. After enzymatic digestion and red blood cell lysis, samples were incubated with an antibody cocktail consisting of: CD4 FITC, CD3 Alexa Fluor 700, CD25 APC/Cy7 (eBioscience, San Diego, CA) and CD8a BUV 737 (BD Biosciences, San Jose, CA). Cells were then permeabilized and incubated with another antibody cocktail consisting of intracellular markers Foxp3 PE, RORγt PerCP/Cy5.5, T-bet Alexa Fluor 647 (BD Biosciences, San Jose, CA) and GATA-3 PE/Cy7 (R&D Systems, Minneapolis, MN). Cells were analyzed by FACS to determine T-cell subsets in the lymph nodes.

### Statistical Analyses

Flow cytometric analyses were carried out using FlowJo Data Analysis Software v10.0 (Tree Star Inc, OR). All other data were analyzed using GraphPad Prism version 6.00 (GraphPad Software, La Jolla, CA). Unpaired student’s t test was used to determine differences between groups. Multiple group comparisons were made using one-way ANOVA with Tukey’s post-hoc tests. Values are expressed as means ± SEM. A value of p < 0.05 was considered significant.

### Data Availability

The datasets generated during and/or analyzed during the current study are available from the corresponding author on reasonable request.

## Conclusions

In conclusion, we report that TREM-2 is expressed on all subsets of dendritic cells in the airways and is upregulated after allergen sensitization and challenge with ovalbumin. This upregulation correlated with increased CCR-7 expression which plays a critical role in migration of DCs to secondary lymphoid organs as well as increased mRNA expression of Th2 and Th17 cytokines. Based on increased expression of CCR-7 and costimulatory molecule CD86 on DC subsets in the draining lymph nodes, the receptor could potentially be playing a role in survival, migration and/or maturation of DC subsets. More in depth studies are however required to confirm this. The findings of this study highlight a potential role of TREM-2 in allergic airway inflammation and the receptor might well be a novel target for therapeutic intervention.
